# Supplementary material for: Linking expression and function of Drosophila type-I TGF-β receptor baboon isoforms: Multiple roles of BaboA isoform in shaping of the adult central nervous system
Source: PLoS One. 2025 May 30;20(5):e0318406. doi: 10.1371/journal.pone.0318406 (PMC12124520; doi:10.1371/journal.pone.0318406)
Supplement: S1 Table — (DOCX) [file pone.0318406.s001.docx]

S1 Table. Pairs of primers to produce C-terminal and isoform-specific Babo-EGFP conjugation, and primers to validate transgenic fly lines.

| primer | usage | Sequence (5’ > 3’) |
| --- | --- | --- |
| baboCtermfor | recombineering | CATCAAAAAGACACTTGCTAGCATCAGTGTGGAGGACAAGGTCAAGAACGCAGCCCAATTCCGATCATATTC |
| baboGFPrev2 | recombineering | CGATCCCACGGGGCGTGACACGAGGGCTACTCGTCAAACTGAAGCCACAATCATTACTTGTACAGCTCGTCCATG |
| baboAGFPfor | recombineering | CGTATAGGATTGATTGCTGTAAGAGTGATTTCTGCAATAAGAATGAGATTATGGTGAGCAAGGGCGAGGAG |
| baboAGFPrev | recombineering | GTTTTTGTTACTTGAATGCGGCTGTACCTGTTTCAAATATCCTCTTCATACTAGTGGATCCCCTCGAGGGAC |
| baboBGFPfor | recombineering | CAAAGACGAAGGACCAGAGGAGTGATCGTGTCGAAGAAGGGCGGCAAATCATGGTGAGCAAGGGCGAGGAG |
| baboBGFPrev | recombineering | GGGGTTTTTGGGAGCGAAACACGTGCATAAGCGCACGCACCTTGGACGGAACTAGTGGATCCCCTCGAGGGAC |
| baboCGFPfor | recombineering | CTTCTCAGTGCTGCGCCGAAGATTTTTGCAATACGCGTGAGAATTACAGTATGGTGAGCAAGGGCGAGGAG |
| baboCGFPrev | recombineering | GAATTGTTGGGCGGTGTTGGCGGTGCATTGCTGGTCTCACCTGGTAGGACACCACTAGTGGATCCCCTCGAGGGAC |
| NEGFPfor | gPCR, seq | ctcgtgaccaccctgacc |
| BaboAflankR | gPCR | ggagtgcaaactaaagatag |
| BaboBflankR | gPCR | ctggttgttgaacggcg |
| BaboCflankR | gPCR | ggtgcaatggcatgggcg |

For recombineeering primers, *babo* tails are shown in red, and plasmid sequences are in black--*C term PL452* *for* and *C EGFP* for C-terminal construct; *N term PL452 for* and *N EGFP rev* for isoform-specific constructs. Primers for gPCR were used to confirm that transgenic fly lines harbored the expected construct. NEGFPfor was paired with flankR primers for each isoform to amplify a specific engineered junction from genomic DNA of transgenic fly stocks. NEGFPfor was used to sequence the PCR products.
