# Supplementary material for: Linking expression and function of Drosophila type-I TGF-β receptor baboon isoforms: Multiple roles of BaboA isoform in shaping of the adult central nervous system
Source: PLoS One. 2025 May 30;20(5):e0318406. doi: 10.1371/journal.pone.0318406 (PMC12124520; doi:10.1371/journal.pone.0318406)
Supplement: S1 File — S1 Fig. Confirmation and exposition of in-frame GFP fusions of Baboon isoform transgenic fly lines. (A) Amplification of specific GFP-Babo intron junctions from genomic DNA of isoform-GFP stocks. PCR products from the indicated combinations of primer pairs and templates were resolved on an agarose gel. Expected junction products were obtained for each isoform tag (bracket at the right of the gel): ‘a’ reverse primer on A template (Lane 1; expected size 691 bp; ‘b’ reverse primer on B template (Lane 5; expected size 701 bp); ‘c’ reverse primer on C template (Lane 9; expected size 706). Other lanes show the specificity of the amplification, and in some cases longer products representing introns downstream of the GFP insertion (Lanes 2, 7, and 8; size range shown by curly brace to the right of the gel). L represents ladder with representative fragment sizes indicated. (B) Deduced amino acid sequence for GFP-isoform junctions. Specific PCR products were sequenced to confirm that the transgenic fly stocks have GFP in-frame with the Babo exons for the ABC isoforms. Translation of the relevant portion of the nucleotide sequence for each isoform shows the C-terminus of GFP (bold), 20 amino acids from the recombineering vector (italics), and the several downstream amino acids encoded by Babo exon 4A/B/C. The highlighted ‘g’ following the last complete isoform-specific codon contributes to the common D residue after splicing. The first seven nucleotides of isoform-specific intron 4 are shown in red in lower case. S2 Fig. Expression of GFP-tagged Babo isoforms in the larval muscle. (A) Detection of indicated GFP-tagged isoforms in the muscle. Specimen showing very low expression levels of individual isoforms only in the cytoplasm (a-c) and relatively strong expression at the NMJ indicated by arrows (a’-c’). (B) Larval body wall muscle expressing pan-Babo-GFP (a-a’) and Mef2-Gal4-driven EGFP (b) at the NMJ (arrows). The postsynaptic NMJ marked by Dlg are shown in (Ba’). Scale b [file pone.0318406.s002.pdf]

S1 Fig

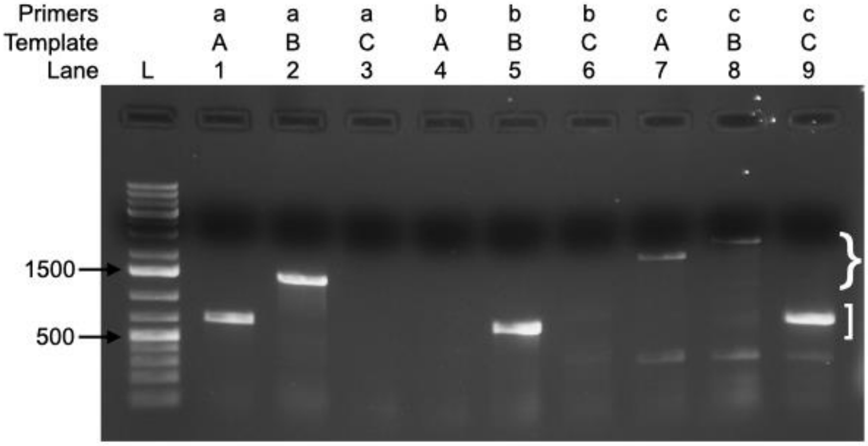

Babo-A-GFP

ATCACTCTCGGCATGGACGAGCTGTACAAGATAACTTCGTATAATGTATGCTATACGAAGTTATTAGGTCCCTCG

I..T..L..G..M..D..E..L..Y..K.. *I..T..S..Y..N..V..C..Y..T..K..L..L..G..P..S..*

AGGGGATCCACTAGTATGAAGAGGATATTTGAAACAGgtacagc

*R..G..S..T..S..*M..K..R..I..F..E..T..[intron 4A]

Babo-B-GFP

ATCACTCTCGGCATGGACGAGCTGTACAAGATAACTTCGTATAATGTATGCTATACGAAGTTATTAGGTCCCTCG

I..T..L..G..M..D..E..L..Y..K.. *I..T..S..Y..N..V..C..Y..T..K..L..L..G..P..S..*

AGGGGATCCACTAGTTCCGTCCAAGgtgcgtg

*R..G..S..T..S..*S..V..Q..[intron 4B]

Babo-C-GFP

ATCACTCTCGGCATGGACGAGCTGTACAAGATAACTTCGTATAATGTATGCTATACGAAGTTATTAGGTCCCTCG

I..T..L..G..M..D..E..L..Y..K.. *I..T..S..Y..N..V..C..Y..T..K..L..L..G..P..S..*

AGGGGATCCACTAGTGGTGTCCTACCAGgtgagac

*R..G..S..T..S..*G..V..L..P..[intron 4C]

S2 Fig

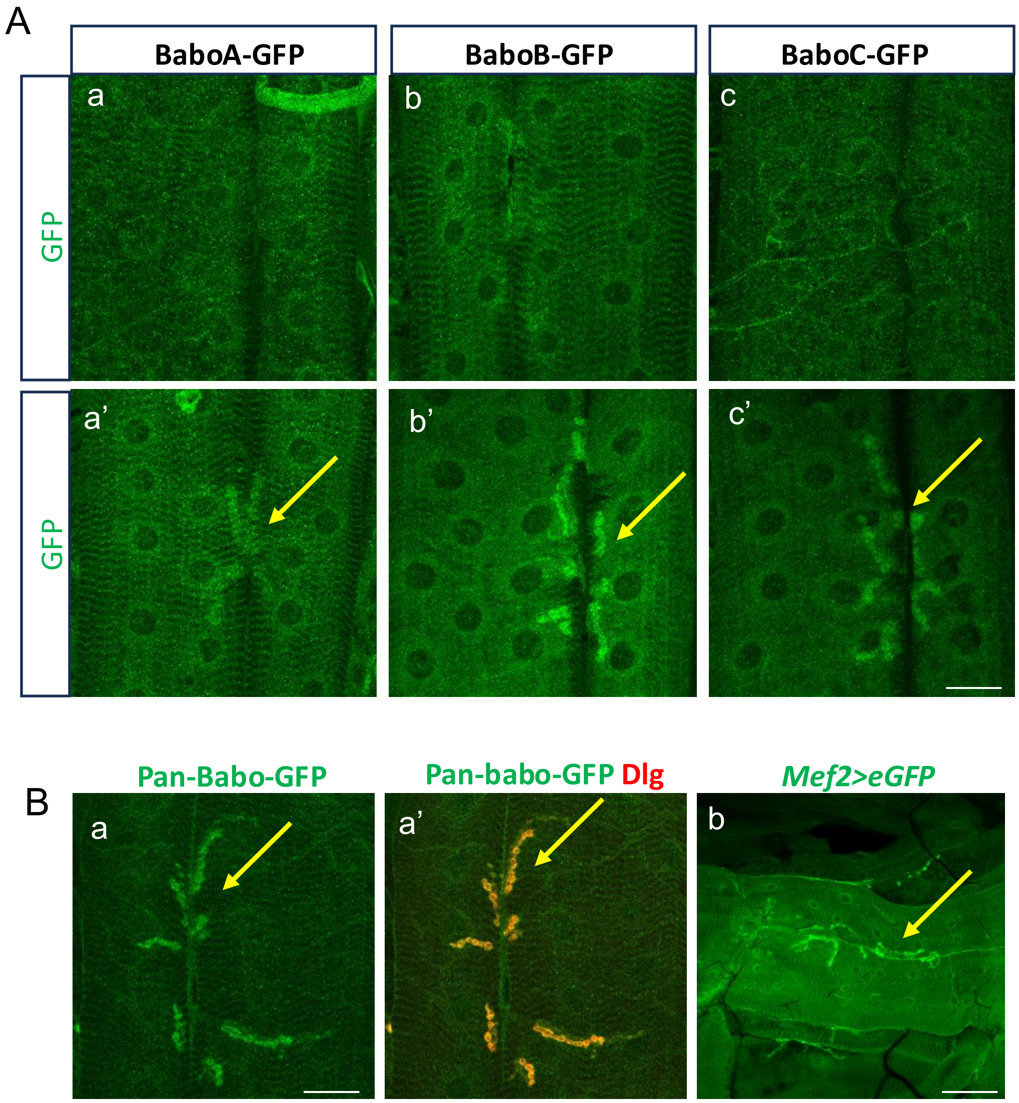
A
BaboA-GFP
BaboB-GFP
BaboC-GFP
GFP
a
b
c
GFP
a'
b'
c'
B
Pan-Babo-GFP
Pan-babo-GFP Dlg
Mef2>eGFP
a
a'
b

S3 Fig

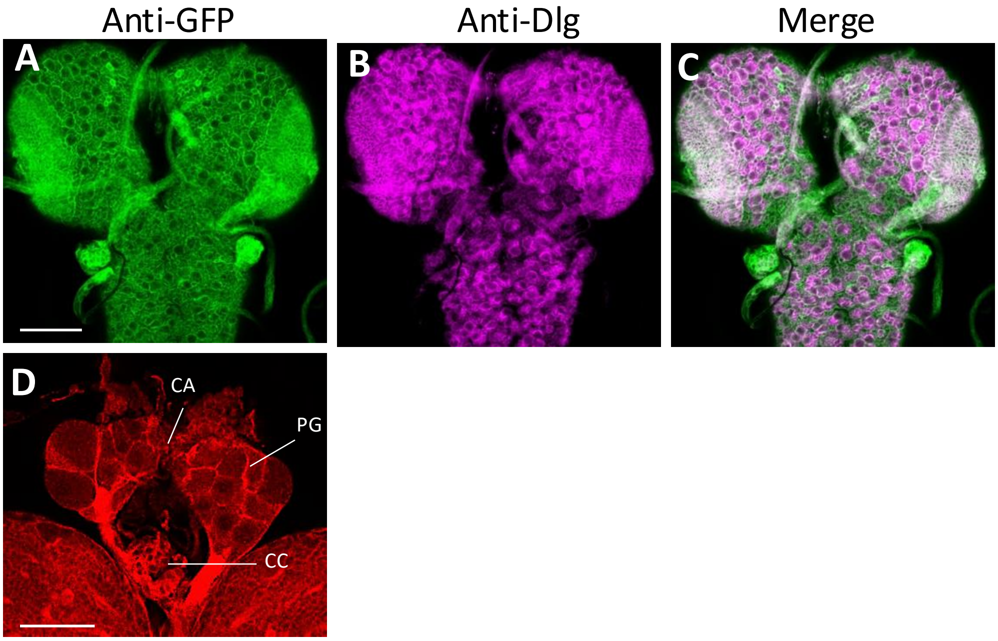

S4 Fig

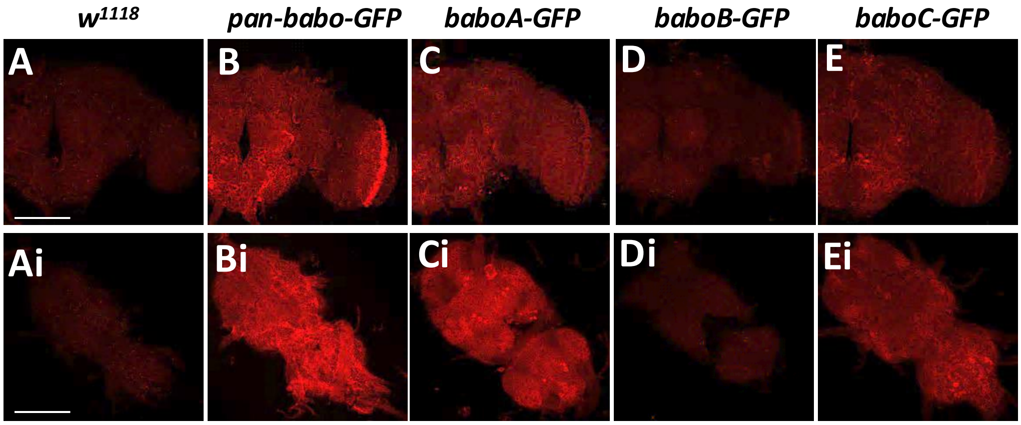

w1118
pan-babo-GFP
baboA-GFP
baboB-GFP
baboC-GFP
A
B
C
D
E
Ai
Bi
Ci
Di
Ei

S5 Fig

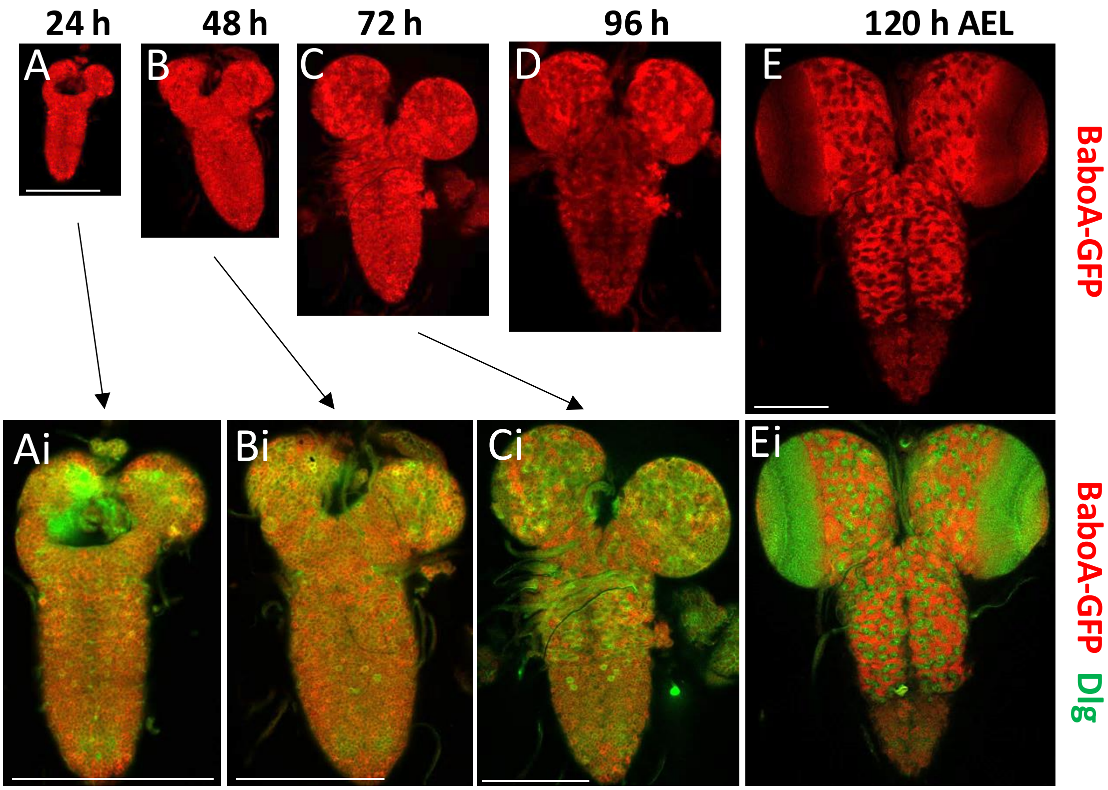

24 h
48 h
72 h
96 h
120 h AEL
A
B
C
D
E
Ai
Bi
Ci
Ei
BaboA-GFP
BaboA-GFP Dlg

S6 Fig

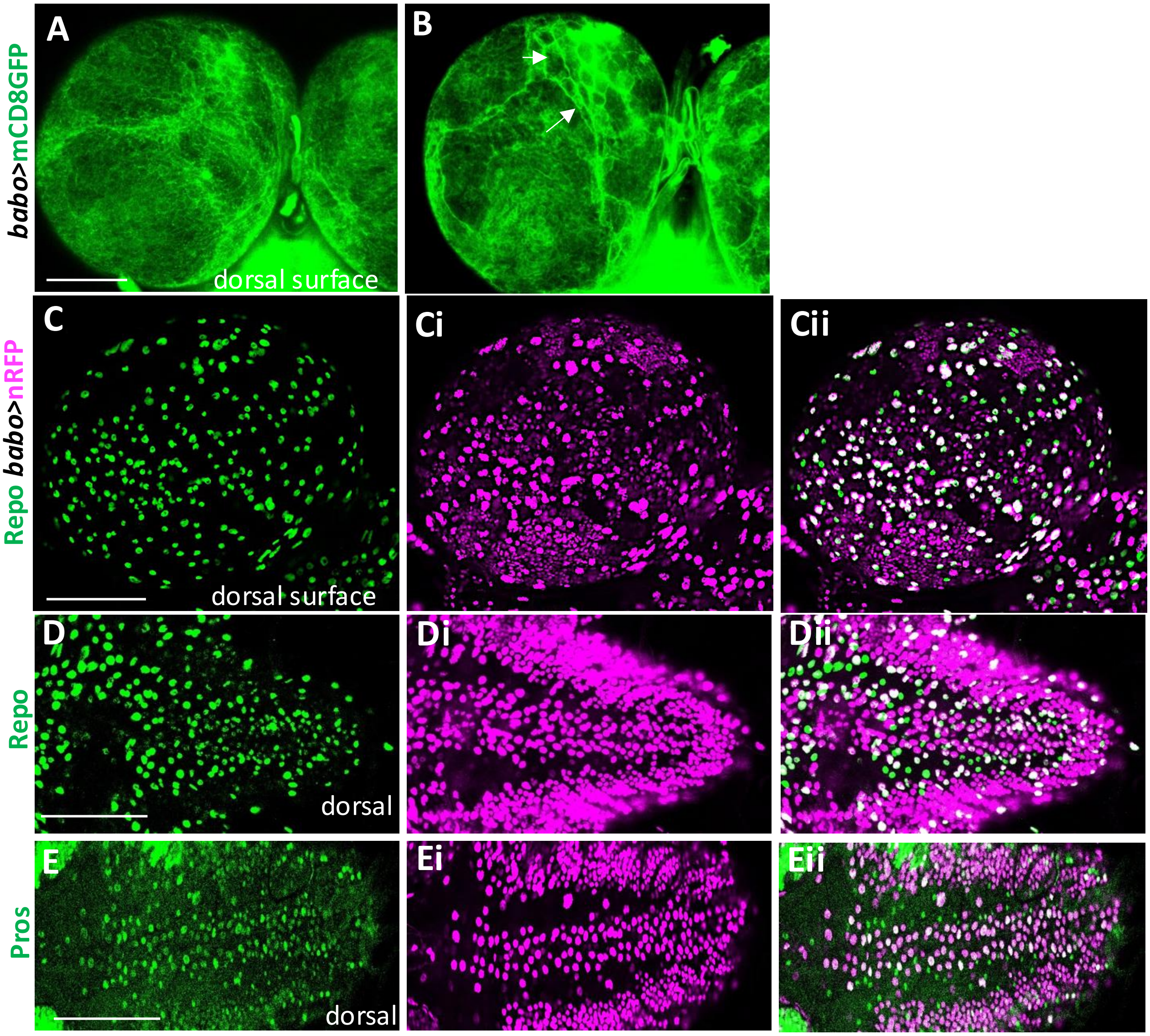

A
B
babo>mCD8GFP
dorsal surface
C
Ci
Cii
Repo babo>nRFP
dorsal surface
D
Di
Dii
Repo
dorsal
E
Ei
Eii
Pros
dorsal
